# Comparison of a 3D multi‐group $S_{N}$ particle transport code with Monte Carlo for intercavitary brachytherapy of the cervix uteri

**DOI:** 10.1120/jacmp.v11i1.3103

**Published:** 2009-12-03

**Authors:** Kent A. Gifford, Todd A. Wareing, Gregory Failla, John L. Horton, Patricia J. Eifel, Firas Mourtada

**Affiliations:** ^1^ Division of Radiation Oncology The University of Texas M.D. Anderson Cancer Center Houston TX USA; ^2^ Transpire Inc. Gig Harbor WA USA

**Keywords:** deterministic calculation, brachytherapy, Monte Carlo, ovoids, C137s

## Abstract

A patient dose distribution was calculated by a 3D multi‐group $S_{N}$ particle transport code for intracavitary brachytherapy of the cervix uteri and compared to previously published Monte Carlo results. A Cs‐137 LDR intracavitary brachytherapy CT data set was chosen from our clinical database. MCNPX version 2.5.c, was used to calculate the dose distribution. A 3D multi‐group $S_{N}$ particle transport code, Attila version 6.1.1 was used to simulate the same patient. Each patient applicator was built in SolidWorks, a mechanical design package, and then assembled with a coordinate transformation and rotation for the patient. The SolidWorks exported applicator geometry was imported into Attila for calculation. Dose matrices were overlaid on the patient CT data set. Dose volume histograms and point doses were compared. The MCNPX calculation required 14.8 hours, whereas the Attila calculation required 22.2 minutes on a 1.8 GHz AMD Opteron CPU. Agreement between Attila and MCNPX dose calculations at the ICRU 38 points was within ±3%. Calculated doses to the 2 cc and 5 cc volumes of highest dose differed by not more than ±1.1% between the two codes. Dose and DVH overlays agreed well qualitatively. Attila can calculate dose accurately and efficiently for this Cs‐137 CT‐based patient geometry. Our data showed that a three‐group cross‐section set is adequate for Cs‐137 computations. Future work is aimed at implementing an optimized version of Attila for radiotherapy calculations.

PACS number: 87.53.Jw

## I. INTRODUCTION

Intracavitary brachytherapy (ICBT) combined with external beam irradiation for treatment of cervical cancer is highly successful in achieving local control. The University of Texas M.D. Anderson Cancer Center employs Fletcher Suit Delclos applicators for low‐dose rate intracavitary brachytherapy. The Fletcher Suit Delclos ovoids contain tungsten alloy shields to limit dose to the bladder and rectum. Most modern treatment planning systems do not explicitly account for the shields in the dose calculation.

The Monte Carlo method has been employed to calculate the effect of the shields on the dose distributions surrounding the ovoids^(1)^ as well as patient geometries.^(2)^ In fact, it has been shown that the ovoids can reduce the dose by as much as 50%.^(^ ^1^ ^,^ ^3^ ^)^ However, Monte Carlo can be computationally prohibitive. Without variance reduction schemes, suitable approximations, or extensive computing resources, the Monte Carlo method cannot calculate the dose distributions in a clinically acceptable time frame.

An alternative approach is to deterministically solve the Boltzmann transport equation (BTE). Attila^(4)^ (Transpire Inc., Gig Harbor, WA), an efficient, generalized geometry transport code developed at Los Alamos National Laboratory, solves the three‐dimensional linear BTE. Attila solves the BTE by discretizing all variables, energy (multi‐group method), space (finite element), and angle (discrete ordinates or $S_{N}$ method) and then iteratively solving the differential form of the BTE for the phase space solution everywhere in the computational domain.

The deterministic method has been applied to brachytherapy calculations.^(^ ^5^ ^–^ ^9^ ^)^ All of the aforementioned studies calculated dose distributions around a single source or applicator, except Zhou et al.^(9)^ Zhou et al. parallelized their implementation of the deterministic method and calculated dose resulting from an 81 seed I125 prostate implant. Previously,^(7)^ we calculated the dose distribution around a Fletcher Suit Delclos ovoid loaded with Selectron Cs‐137 pellets (Nucletron Trading BV, Veenendaal, The Netherlands) in a water phantom with a nine energy group cross‐section set and $S_{18}$ angular order. The goal of this study was to benchmark the Attila code in the presence of many heterogeneities (ICBT tandem and ovoid applicators and their constituents) with a three‐group energy cross‐section set and reduced angular order. These calculations will be compared to an identical patient case calculated previously^(2)^ by MCNPX.

## II. MATERIALS AND METHODS

### A. Patient selection and scanning

A case was selected from an Investigational Review Board (IRB) approved institutional trial. Each patient receiving low‐dose rate ICBT typically undergoes two insertions. In this study, the patient dose distribution was calculated for one insertion. Total doses from two ICBT implants and a course of external beam radiotherapy (EBRT) are difficult to ascertain because of the differences in the biological responses between ICBT and high‐dose rate EBRT, the different fractionation schemes of ICBT and EBRT, and the deformation and displacement of structures irradiated by the two modalities.^(10)^

CT scans were performed on an AcQSim CT simulator (Philips Medical Systems, Andover, MA). Scans were acquired in helical mode at 120 kVp, 250 mA with a slice thickness of 3 mm. Each image was 512×512 pixels with a 12‐bit pixel depth. The Foley bulb was filled with 7 mL of a solution that contained three parts Hypaque contrast and seven parts saline. Just prior to scanning, 20 mL of a more dilute solution containing 3 mL of Hypaque and 17 mL of saline was instilled in the bladder. Patients were scanned supine immediately after placement of the tandem and ovoids. A specially fabricated insert marking the position of each pellet inside each applicator was placed in the tandem and ovoids prior to scanning. Images were transferred to the BrachyVision treatment planning system (Varian Medical Systems, Concord, CA) for segmentation, source delineation and planning.

Once transferred to the planning system, the entire bladder was contoured. The rectum was contoured from the bottom of the ischial tuberosities to the sigmoid flexure. Each slice of segmented image data was exported in bitmap format. The ${\text{Pinnacle}}^{3}$ version 6.2b treatment planning system (Phillips Medical Systems, Milpitas, CA) was used as a display device, as well as to calculate dose to points in the bladder and rectum. An IDL program (Research Systems Inc., Boulder, CO) was written to read in all of the exported bitmap files and convert them to ${\text{Pinnacle}}^{3}$ image format. An image header file was created so that the ${\text{Pinnacle}}^{3}$ treatment planning system would recognize the image file. ${\text{Pinnacle}}^{3}$ did not have the coordinates of points on each contour from the exported bitmap files; therefore, these contours were traced in ${\text{Pinnacle}}^{3}$.

A MATLAB program (The Mathworks Inc., Natick, MA) was written that determined the position and orientation of each applicator in the patient CT scan. The program output transformation matrices for the tandem and ovoids for the patient. These transformation matrices moved the applicators to their appropriate positions for radiation transport.

### B. Patient treatment

All patients included in the IRB approved institutional trial were treated with the Selectron remote afterloading LDR unit coupled with the Fletcher Suit Delclos (FSD) tandem and ovoids for cervical cancer brachytherapy treatments. Three channels are utilized for patient treatment – one for the tandem and one each for the right and left ovoids. Each channel contains 48 pellets. The sources (active pellets) and inactive pellets are remotely afterloaded into the tandem and ovoids after the staff have evacuated the room.

Patients that receive an ICBT insertion are generally under either spinal or general anesthesia. After a recto‐vaginal examination, the external os is located and two radio‐opaque markers are placed in the os. One is inserted anteriorly, while the other is inserted posteriorly. To determine the intrauterine length of the tandem, a uterine sound is inserted in the uterus. A flange is placed on the tandem after insertion, which abuts the cervical os. This flange prevents cephalad motion of the tandem.

The ovoids are positioned within the vaginal vault in the fornices. The largest size ovoid is selected so the maximum possible distance between sources and vaginal mucosa is achieved. Plastic caps can be placed over the small ovoids to create either a medium or large ovoid, if necessary. After positioning of the ovoids, gauze packing lined with a radio‐opaque wire is packed about the tandem and ovoid system. This limits movement of the system and, if correctly placed, reduces the dose to the bladder and rectum.

### C. Monte Carlo simulation

The Monte Carlo code MCNPX (Monte Carlo *N*‐Particle) version 2.5.c^(11)^ was used to perform the simulations in this study. MCNPX is a general‐purpose Monte Carlo code that can transport 34 types of particles and more than 2000 heavy ions. MCNPX includes many convenient features such as a powerful geometry modeling tool and various tallies related to energy deposition, particle current and particle flux. Photons and electrons can be transported in a range of energies of 1 keV to 1000 MeV. Photon transport includes the photoelectric effect, coherent scattering, Compton scattering, and pair production. Characteristic X‐ray production accompanying the photoelectric effect can also be simulated. Greater detail concerning the code can be found elsewhere.^(11)^

Water photon kerma rates were calculated by MCNPX. Charged particle equilibrium was assumed to exist so that the dose could be approximated as collision kerma. The simulation geometry included the applicator casings, internal constituents, as well as the Cs‐137 pellets. Hence, the resultant dose matrix included the effects of each applicator and their attenuation, in addition to inter‐applicator attenuation. The Cartesian dose grid was determined by inspecting the positions of points A and B, and the extent of the bladder and rectum from the patient CT scan. A sphere of water 25 cm in radius surrounded the dose grid. The voxel size for the patient case was $2\times 2\times 3\;{\text{mm}}^{3}$. Twenty million histories were simulated so that the maximum overall relative error was approximately ±2%(k=1) at a point 5 cm lateral to the ovoids. A reference geometry was created in an input file that allowed each applicator to be positioned in a patient CT‐derived geometry by simply applying a transformation obtained from a MATLAB program. Further details may be found elsewhere.^(2)^

### D. Attila simulation

Attila version 6.1.1^(12)^ was used to perform the calculations in this study. Attila is a radiation transport system that has been applied to several areas, including nuclear power, homeland security, imaging systems, sterilization, waste storage, oil exploration, satellite design, radiological safety, and non‐invasive materials. Attila solves the three‐dimensional linear steady state Boltzmann transport equation. This equation is given below:

(1) $$\frac{d}{ds}\psi (\vec{r},E,\hat{\Omega })+\sigma_{t}(\vec{r},E)\psi (\vec{r},E,\hat{\Omega })=Q_{s}(\vec{r},E,\hat{\Omega })+q(\vec{r},E,\hat{\Omega })$$

where, ψ is the angular fluence rate $(\text{particles}•{\text{cm}}^{2}•\text{steradian}•\text{second}•\text{MeV}),\;d/\text{ds}$ is the directional derivative along the particle flight path, $\hat{\Omega }$ is the unit vector denoting the particle direction, $\sigma_{t}$ is the total macroscopic interaction cross section $\left({\text{cm}}^{-1}\right)$, $\sigma_{s}$ is the differential macroscopic scattering cross section ${(\text{cm}}^{-1}•{\text{steradian}}^{-1}•{\text{MeV}}^{-1})$, and *q* is the fixed source $\left(\text{particles}•{\text{cm}}^{-3}•\text{steradian}•\text{second}•\text{MeV}\right)$. The scattering source, $Q_{s}$, is given below:

(2) $$Q_{s}(\vec{r},E,\hat{\Omega })=\int_{0}^{\infty }\int_{4\pi }\sigma_{s}(r,E^{\prime }\to E,\hat{\Omega }\cdot \Omega^{\prime })\psi (r,E^{\prime },{\hat{\Omega }}^{\prime })\;d{\hat{\Omega }}^{\prime }dE^{\prime }$$

Attila employs multi‐group energy discretization, discrete‐ordinates in angle $\left(S_{n}\right)$, and linear discontinuous finite element differencing spatially to solve the LBTE. The resulting solution is a particle distribution function in space, angle and energy that can be modified to produce user defined edits such as dose.

The general solution technique is source iteration (SI). SI is a simple and effective method for many classes of transport problems. However, for problems dominated by scattering, the SI method can converge slowly. The method known as diffusion synthetic acceleration (DSA) has been shown to dramatically decrease the number of source iterations required for convergence. Here, a diffusion approximation is used to approximate the error in the transport SI process. Attila uses a simplified DSA method to accelerate the source iteration process. This method has been shown to be unconditionally stable and highly effective in the vast majority of practical transport problems. Further details can be found elsewhere.^(^ ^4^ ^,^ ^7^ ^)^

The tandem and ovoids were modeled in SolidWorks (Solidworks Corp,Concord, MA), a mechanical design automation package. Each applicator was built in SolidWorks and then assembled with a coordinate transformation and rotation derived previously. SolidWorks exports applicator geometries as a Parasolid geometry. This geometry file is then imported into the Attila code for calculation. After importing the Parasolid geometry file, a calculation mesh is constructed by entering the maximum length of the side of a tetrahedron for each region of the geometry. A mesh is then constructed based on these inputs. This feature allows the user to refine the grid based on parts of the geometry that are important. Attila solves the BTE at each corner of each tetrahedron.

The calculation grid was equivalent to those used in the MCNPX calculations. The Attila calculations were performed with $S_{12}$ (168 ordinates), $P_{2}$ scattering order, and three energy groups. The three photon energy groups were: 0.7‐0.6, 0.6‐0.2, and 0.2‐0.001 MeV. The first scatter distributed source technique was employed to mitigate ray‐effects. Each of the first scatter distributed sources was located at the center of the Selectron Cs‐137 pellets in the tandem and ovoids for a total of 13 sources. Similar to the MCNPX calculation, inter‐ and intra‐applicator attenuation and scattering were explicitly accounted for in the simulation.

### E. Data analysis and comparison

Water photon kerma rate was converted to dose delivered for the duration of one insertion. The tandem contained seven sources for a total of $231\;U\;(\mu \text{Gy}\;m^{2}\;{\text{hr}}^{-1})$. Each ovoid contained three sources and, collectively, the air kerma strength was $198\;U\;(\mu \text{Gy}\;m^{2}\;{\text{hr}}^{-1})$. The total reference air kerma for this patient insertion was 19200 μGy at 1 m. All calculations were performed on a 1.8 GHz AMD Opteron computer. The dose matrices from the MCNPX and Attila runs were formatted in an IDL code so that they could be imported into the ${\text{Pinnacle}}^{3}$ treatment planning system ver 6.2b for analysis. Dose volume histograms for the bladder and rectum were overlaid and exported. The ICRU 38 points were also compared. Representative transverse slices were also exported for qualitative comparison.

## III. RESULTS & DISCUSSION

**Figure 1:**
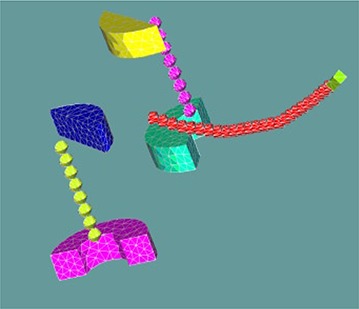
A 3D representation of the patient geometry. Portions of the geometry were intentionally omitted to allow visualization of the shields, pellets, and tandem tip screw.

Figure 1 displays the calculation mesh for the patient geometry. Portions of the geometry were omitted from this figure so that the internal constituents of the ovoids and tandem could be visualized. The patient calculation mesh contained 119,637 cells.

**Figure 2:**
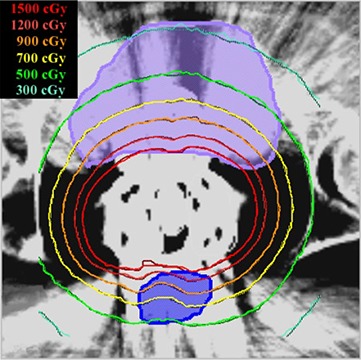
Transverse slice from patient dose distribution. The bladder is shown in lavender; the rectum in blue. MCNPX‐calculated values are indicated by solid lines; the Attila‐calculated values by dashed lines.

Figure 2 illustrates a dose comparison within a transverse plane between the two codes. For problems with localized source (near point sources) in weakly scattering media (soft tissue), anomalies in the solution, known as “ray‐effects” arise. This results in nonphysical oscillations in the solution due to a nonphysical buildup in the fluence rate along the discrete angles. Attila incorporates an analytic ray tracing method in order to mitigate these ray‐effects. This patient case included 13 first‐scatter distributed point sources located at the physical center of each active Cs‐137 pellet. It is apparent that the 13 first‐scatter distributed point sources coupled with $S_{12}$ angular order is sufficient to alleviate ray‐effects and provide an accurate solution as compared to the MCNPX results.

**Figure 3:**
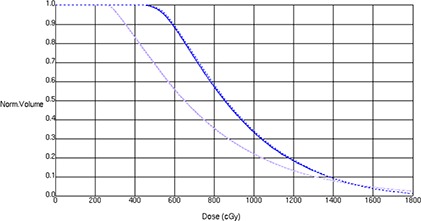
Patient bladder and rectal DVHs. The bladder DVH is indicated in lavender; the rectal DVH in blue. MCNPX calculations are indicated by solid lines; the Attila calculations by dashed lines.

Figure 3 is a DVH comparison for the bladder and rectum. The mean percent difference between MCNPX and Attila for the bladder DVH is 1.4%. The mean percent difference between MCNPX and Attila for the rectal DVH is 1.8%. These values are within the statistical uncertainty of the MCNPX results. Table 1 shows the dose at each ICRU 38 point and the percent difference between Attila and MCNPX calculated values. Table 2 lists the doses to the 2 cc and 5 cc volumes of highest dose to the bladder and rectum as well as the percent difference between the estimations of the two codes. The differences between the two codes in Tables 1 and 2 are within the statistical uncertainty of the MCNPX simulated values. The MCNPX simulation required 14.8 hours of computing time, whereas the Attila simulation required 22.2 minutes.

**Table 1:** Comparison of dose at each of the ICRU 38 reference points, calculated by MCNPX and Attila. MCNPX uncertainties are quoted at 1σ.

	*MCNPX (cGy)*	*Attila (cGy)*	*% diff*
Art	2213 ± 22	2223	0.5
Alt	2193 ± 22	2166	−1.2
Brt	680 ± 12	681	0.1
Blt	598 ± 11	597	−0.2
Bladder	1455 ± 18	1472	1.2
Rectum	952 ± 14	980	2.9

**Table 2:** Comparison of dose at the 2 cc and 5 cc volumes of highest dose calculated by MCNPX and Attila for both rectum and bladder: $D_{\text{RV}2}$ is the dose to the 2 cc volume of highest dose for the rectum; $D_{\text{RV}5}$ is the dose to the 5 cc volume of highest dose for the rectum; $D_{\text{RV}2}$ is the dose to the 2 cc volume of highest dose for the bladder; $D_{\text{RV}5}$ is the dose to the 5 cc volume of highest dose for the bladder.

	*MCNPX (cGy)*	*Attila (cGy)*	*% diff*
$D_{\text{RV}2}$	1526	1526	0.0
$D_{\text{RV}5}$	1294	1298	−0.3
$D_{\text{RV}2}$	1929	1908	1.1
$D_{\text{RV}5}$	1687	1670	1.0

## IV. CONCLUSIONS

This study demonstrated that Attila accurately calculates dose as compared to MCNPX for Cs‐137 based ICBT. In particular, we modeled the Cs‐137 spectrum with a three group cross‐section set. Several authors have modeled photon spectra with a few group cross section set. Gifford et al.^(13)^ demonstrated for a commercially available Ir‐192 HDR source that a five‐group cross‐section set was as accurate as a 15‐group cross‐section set, yet more efficient. Daskalov et al.^(6)^ collapsed the I‐125 spectrum into three groups, and calculated the AAPM TG‐43 parameters for a single 6702 brachytherapy source. They concluded that a three‐group cross‐section set is dramatically more efficient than a many group cross‐section set yet just as accurate. However, their three‐group set required the use of a weighting function. There was no such use of weighting functions in the generation of our three‐group cross‐section set.

Attila can calculate dose in a Cs‐137 LDR CT based patient geometry in less than a half hour. This fact has important ramifications for brachytherapy treatment planning, as commercially available brachytherapy treatment planning systems are incapable of calculating dose in the presence of heterogeneities. Attila and MCNPX are generalized transport codes and neither has been optimized for radiotherapy transport. Yet, discrete ordinates‐based codes have been shown to transport photons through arbitrary geometries more efficiently than other general Monte Carlo transport codes.^(14)^ A radiotherapy‐optimized version of Attila could calculate treatment plans in a clinical time frame.

A limitation of this study is that we did not model the female pelvis explicitly. Rather, we modeled the pelvis homogeneously. This fact does not detract from the results, as no calculation points were located in a region where heterogeneities would affect the dose determination. However, Attila is ideally suited to handle heterogeneous geometries.^(^ ^7^ ^,^ ^15^ ^)^ An interesting application of this technology would be in partial breast brachytherapy, particularly MammoSite (Hologic Inc., Bedford, MA). Many MammoSite patients present with air pockets and/or seroma volumes as well as having radiographic contrast present in the balloon. These volumes as well as the lung and ribs are not explicitly accounted for by most commercially available treatment planning systems.^(16)^ Perhaps the implementation of Attila or a suitable radiotherapy optimized variant would improve the ability to predict dose and ultimately dose response studies.

There are several features which could improve the performance of Attila for brachytherapy dose calculations. For example, a linear Ir‐192 source, where attenuation along the major axis presents significant anisotropy, can be modeled in one calculation and stored as an anisotropic point source. This anisotropic point source can be used in subsequent calculations without having to transport through the source materials and encapsulation, saving valuable computational time. Application of this and other novel functions to radiotherapy transport will be the subject of future work.
